# Association between internet addiction and mindfulness

**DOI:** 10.3389/fpsyg.2026.1810492

**Published:** 2026-05-08

**Authors:** Ayumu Kubaru, Hiroki Murakami

**Affiliations:** Department of Psychology, Oita University, Oita, Japan

**Keywords:** acting with awareness, internet addiction, mindfulness, non-judging, observing

## Abstract

**Introduction:**

Studies have shown that mindfulness is negatively associated with Internet addiction. However, how mindfulness reduces Internet addiction remains unclear. We examined the relationship between individual mindfulness facets and Internet addiction.

**Methods:**

We used the Japanese version of the Internet Addiction Test and the Japanese version of the Five Facet Mindfulness Questionnaire (FFMQ) and investigated the association between Internet addiction tendency and mindfulness traits in undergraduate and graduate students (N = 134).

**Results:**

Correlation analyses indicated that the total FFMQ score and the non-judging and acting with awareness facets were negatively associated with Internet addiction tendency. Furthermore, we conducted a multiple regression analysis with all five mindfulness facets as independent variables, in which only acting with awareness was identified as a significant negative predictor of Internet addiction tendency.

**Conclusion:**

These results suggest that more effective interventions for Internet addiction may be achieved by focusing training on non-judging and acting with awareness.

## 1 Introduction

The spread of Internet access has led to structural changes across diverse domains, including interpersonal communication (Li et al., 2022), education (Ren et al., 2024), and work styles (Gibbs et al., 2023). Moreover, wide-ranging online use may alter individuals’ behavioral patterns by affecting attention, memory, and social cognition (Firth et al., 2019). In a society where Internet use has become deeply embedded in daily life, Internet addiction has emerged as a serious public health concern (Young, 1998). Recent research reported that 24.3% of college students in Asia met criteria for Internet addiction and further indicated that Internet addiction is associated with negative physical and emotional health outcomes among college students (Duc et al., 2024).

Recent research has developed different methods for preventing and reducing addictive behaviors. For example, a recent umbrella review of meta-analyses evaluated interventions for digital addiction, including cognitive behavioral therapy, group counseling, and exercise, and found that the overall certainty of evidence for these approaches remains low (Lu et al., 2025). Among these approaches, mindfulness has received increasing attention (Jia et al., 2025). Mindfulness is defined as “paying attention in a particular way: on purpose, in the present moment, and nonjudgmentally” (Kabat-Zinn, 1994). Mindfulness meditation does not rely on cognitive or behavioral strategies to avoid aspects of experience; it adopts an attitude of acceptance toward painful or unpleasant thoughts and feelings (Bishop et al., 2004). Typical practice begins with attention training on a chosen object, such as breathing or bodily sensations, and gradually expands the scope of attention to include a broader range of experiences, including thoughts and emotions (Kabat-Zinn, 1994; Lutz et al., 2008). Clinical research evidence shows that mindfulness-based interventions contribute to reducing the relapse rate in depression by fostering the participants’ ability to distance themselves from thoughts and emotions (Teasdale et al., 2000). More recently, a transdiagnostic meta-analysis of randomized controlled trials found that mindfulness-based interventions significantly reduce depressive symptoms across a range of mental disorders (Alkan et al., 2025).

Studies have also demonstrated the efficacy of mindfulness for improving or preventing drug addiction and gambling addiction. For example, an 8-week mindfulness-based relapse prevention program conducted for 168 individuals with drug and alcohol use disorders reduced craving and substance use compared with treatment as usual (Bowen et al., 2009). Additionally, a 6-week mindfulness-based relapse prevention program for prisoners decreased substance use cravings (Lyons et al., 2019). These findings suggest that mindfulness-based interventions may strengthen a domain-general neurocognitive resource that can modulate a variety of mechanisms implicated in addiction, including reward processing, cue reactivity, and stress reactivity (Garland and Howard, 2018).

Furthermore, a systematic review has confirmed a moderate improvement from mindfulness interventions for gambling addiction. Mindfulness contributed to reducing gambling urges and financial losses (Maynard et al., 2018). In addition, mindfulness-based approaches for smokers have enhanced self-control abilities, including emotion regulation and stress reduction, after training, regardless of the smoking status (Tang et al., 2016). Neuroimaging findings also indicated that brain regions associated with emotional regulation and self-control, particularly the anterior cingulate cortex and adjacent medial prefrontal cortex, had pre-training hypoactivity in smokers and improved after mindfulness training (Tang et al., 2016).

Consistent with findings from substance use and gambling disorders, recent reviews suggest that mindfulness is a promising approach for managing Internet addiction. For example, mindfulness practices have been shown to help individuals regulate compulsive online behaviors, resist impulsive urges, and reduce related comorbidities such as anxiety and depression (Samanta et al., 2024). In line with these findings, empirical studies have reported a negative association between mindfulness and Internet addiction (Zewude et al., 2024). In a study of young adults, mindfulness moderated the mediating effect of self-forgiveness on the relationship between social exclusion and Internet addiction, suggesting a protective role of mindfulness in problematic Internet use (Arslan and Coşkun, 2022).

Despite these findings, the mechanisms underlying the effect of mindfulness on Internet addiction remain unclear. Calvete et al. (2017) examined the relationship between Problematic Internet Use (PIU) and individual facets of mindfulness. PIU is a multidimensional syndrome characterized by cognitive and behavioral symptoms that lead to negative social, academic, or professional outcomes (Caplan, 2005). Using the Five Facet Mindfulness Questionnaire (FFMQ; Baer et al., 2006), Calvete et al. found that the non-judging, acting with awareness, and observing facets were associated with lower PIU. However, PIU was assessed using the Generalized Problematic Internet Use Scale 2 (Caplan, 2010), which emphasizes cognitive and interpersonal processes underlying Internet use, such as preference for online social interaction and mood regulation. As a result, it remains unclear whether mindfulness directly reduces maladaptive Internet use behaviors or primarily influences the underlying cognitive and emotional processes associated with PIU. The purpose of the present study is to elucidate the relationship between each facet of mindfulness and the tendency toward Internet addiction, to assist in developing mindfulness-based interventions tailored to Internet addiction.

This study used the Internet Addiction Test (IAT; Young, 1998), which focuses on behavioral and symptomatic aspects of addiction. The IAT is modeled on diagnostic criteria for pathological gambling and specializes in directly assessing the severity of clinical addiction symptoms, such as difficulty in controlling usage time and withdrawal symptoms. We examined the relationship between individual facets of mindfulness, assessed using the FFMQ, and Internet addiction, assessed using the IAT.

Based on previous findings (Calvete et al., 2017), we hypothesized that non-judging, acting with awareness, and observing facets of mindfulness would be negatively associated with tendencies toward Internet addiction.

## 2 Methods

### 2.1 Participants

This study employed a cross-sectional design. Based on prior findings (Calvete et al., 2017), a moderate effect size was assumed. A power analysis indicated that, with a significance level of 0.05 and statistical power of 0.80, a minimum sample size of 125 participants was required to detect the expected effect with five predictor variables. Accordingly, data collection was conducted with the goal of reaching at least this sample size. Data were collected between April and June 2025 using self-report questionnaires. Undergraduate and graduate students from Oita University participated (*N* = 134, 61 men, 72 women, and 1 other; *M* age = 19.6 years, *SD* = 1.7). They provided written informed consent before taking part in the study. We conducted this study in accordance with the Declaration of Helsinki. The Ethics Committee of the Faculty of Welfare and Health Science at Oita University approved the study (approval number F240034).

### 2.2 Measures

We used the Japanese version of the FFMQ (Sugiura et al., 2012), originally developed by Baer et al. (2006), to assess the five facets of mindfulness: observing, non-reactivity to inner experience, non-judging of inner experience, describing, and acting with awareness. The FFMQ comprises 39 items rated on a 5-point Likert scale ranging from 1 (*never or very rarely true*) to 5 (*very often or always true*). We also used the Japanese version of the Internet Addiction Test (IAT; National Hospital Organization Kurihama Medical and Addiction Center, 2025), originally developed by Young (1998), to assess Internet addiction tendency. The IAT instructions define Internet use as including all online devices, such as personal computers, mobile phones, smartphones, and game consoles. The IAT consists of 20 items rated on a 5-point Likert scale ranging from 1 (*rarely*) to 5 (*always*). Total scores range from 20 to 100, with higher scores indicating a greater tendency toward Internet addiction.

### 2.3 Data analysis

We calculated Pearson correlation coefficients to examine the associations between demographic data (men = 1, women = 0, excluded other), FFMQ and IAT scores, focusing on the relationships between each mindfulness facet and Internet addiction tendency. In addition, a multiple regression analysis was conducted with the five mindfulness facets as independent variables and IAT scores as the dependent variable to assess the independent contribution of each facet to Internet addiction tendency.

## 3 Results

### 3.1 Descriptive statistics

Table 1 presents the demographic characteristics of the participants, along with the means, standard deviations, and Cronbach’s alpha coefficients for each scale. The sample consisted primarily of young adults, and all measures demonstrated acceptable internal consistency.

**Table 1:** Descriptive statistics.

Variables		Mean	*SD*	Cronbach’s *α*
FFMQ	Observing	24.04	5.97	0.74
	Nonreactivity	20.08	4.54	0.66
	Nonjudging	22.85	6.60	0.85
	Describing	22.81	6.13	0.84
	Acting with awareness	23.97	5.93	0.81
	Total score	113.75	15.43	0.80
IAT		54.53	14.97	0.90

FFMQ, Five facet mindfulness questionnaire; IAT, internet addiction test.

### 3.2 Internet addiction and mindfulness facets

Table 2 presents Pearson correlation coefficients between demographic data, FFMQ scores and IAT scores. Significant negative correlations were observed between IAT scores and the non-judging and acting with awareness facets, as well as the total FFMQ score (*r* = −0.17, *p* = 0.049; *r* = −0.41, *p* < 0.001; *r* = −0.19, *p* = 0.026, respectively). In contrast, the observing facet did not show a statistically significant correlation with IAT scores.

**Table 2:** Pearson correlation coefficients between demographic data, the IAT and the FFMQ scores.

Variables	1	2	3	4	5	6	7	8
1. Sex								
2. Age	0.15^+^							
3. Observing	−0.18*	−0.12						
4. Nonreactivity	0.26**	−0.02	−0.03					
5. Nonjudging	0.11	0.13	−0.29**	0.28**				
6. Describing	0.19*	0.05	0.08	0.37**	0.36**			
7. Acting with awareness	−0.07	−0.08	−0.08	−0.05	0.18*	0.17^+^		
8. Total FFMQ score	0.11	0.01	0.26**	0.53**	0.61**	0.75**	0.48**	
9. IAT	0.15^+^	0.17^+^	0.12	0.03	−0.17*	−0.04	−0.41**	−0.19*

**p < 0.01, *p < 0.05, ^+^*p* < 0.10.

FFMQ, Five facet mindfulness questionnaire; IAT, internet addiction test.

### 3.3 Multiple regression analysis of internet addiction and mindfulness facets

**Figure 1:**
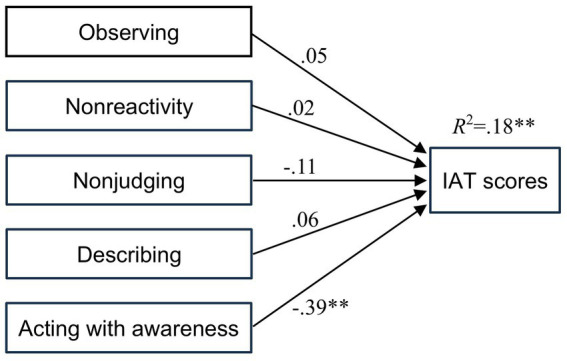
Results of the multiple regression analysis predicting internet addiction test scores from the five facet mindfulness questionnaire. IAT, internet addiction test. **p < 0.01, *p < 0.05, ^+^*p* < 0.10.

To examine the independent contributions of the five mindfulness facets to Internet addiction tendency, we conducted a multiple regression analysis with the five facets as independent variables and IAT scores as the dependent variable. Table 3 and Figure 1 present the results. The overall model was significant and explained 18% of the variance in Internet addiction tendency (*R*^2^ = 0.18, adjusted *R*^2^ = 0.15), *F* (5, 128) = 5.76, *p* < 0.001.

**Table 3:** Multiple regression analysis predicting the IAT scores from FFMQ facets.

Variables	*b*	SE	*β*	*t*	*p*	95% CI	VIF
Observing	0.12	0.21	0.05	0.55	0.58	−0.31, 0.54	1.15
Nonreactivity	0.06	0.29	0.02	0.22	0.83	−0.51, 0.64	1.22
Nonjudging	−0.25	0.21	−0.11	−1.18	0.24	−0.67, 0.17	1.35
Describing	0.14	0.23	0.06	0.61	0.55	−0.31, 0.59	1.35
Acting with awareness	−0.99	0.21	−0.39**	−4.75	<0.001	−1.41, −0.58	1.07
*R* ^2^	0.18**						

**p < 0.01, *p < 0.05, ^+^*p* < 0.10.

FFMQ, Five facet mindfulness questionnaire; IAT, internet addiction test.

The standardized regression coefficients showed that acting with awareness was the only significant negative predictor of Internet addiction tendency (*β* = −0.39, *t* = −4.75, *p* < 0.001). Moreover, non-judging showed a negative association, which did not reach statistical significance (*β* = −0.11, *t* = −1.18, *p* = 0.24). The remaining three mindfulness facets were not significant predictors. Variance inflation factor (VIF) values ranged from 1.07 to 1.35, indicating no evidence of multicollinearity.

## 4 Discussion

We examined the relationship between individual facets of mindfulness and Internet addiction using the FFMQ and the IAT. The total FFMQ score showed a significant negative correlation with Internet addiction tendency. This finding was consistent with previous studies indicating that mindfulness exerts an inhibitory effect on Internet addiction (Zewude et al., 2024). In addition, non-judging and acting with awareness among the five mindfulness facets were negatively associated with Internet addiction tendency. These findings are consistent with previous studies linking non-judging and acting with awareness to reduced Problematic Internet Use (PIU; Calvete et al., 2017) and thus generally support the study hypotheses.

Furthermore, the multiple regression analysis indicated that acting with awareness had a significant negative relationship with Internet addiction tendency even after controlling for the other mindfulness facets. The acting with awareness facet reflects the tendency to attend to one’s current activities and avoid automatic pilot (Baer et al., 2006). The importance of acting with awareness has been repeatedly emphasized in prior research. Calvete et al. (2017), for example, demonstrated that acting with awareness predicts less deficient self-regulation of Internet use, thereby indirectly lowering the risk of PIU. Similarly, Gámez-Guadix and Calvete (2016) reported that higher mindful awareness is associated with lower self-regulation impairments and less severe symptoms of Internet use. Overall, these findings suggest that acting with awareness plays a central role in preventing Internet addiction by enhancing self-regulatory control over impulsive Internet use.

Observing was not significantly associated with Internet addiction tendency in this study, even though Calvete et al. (2017) reported an association between observing and PIU in adolescents aged 11–18 years. This discrepancy may reflect differences in participant age, as the study’s participants were adults. Moreover, PIU captures psychosocial difficulties related to Internet use broadly. In contrast, the IAT (Young, 1998) is based on diagnostic criteria for pathological gambling. It focuses more directly on behavioral and symptomatic aspects of addiction, such as impaired control over usage time and withdrawal symptoms. Therefore, even if observing is related to PIU, it may not be associated with the specific addiction-related behaviors assessed by the IAT.

Previous research indicates that the function of the observing facet varies depending on meditation experience and individual psychological characteristics (Baer et al., 2006, 2008). Observing is not consistently associated with acceptance-related facets such as non-judging or non-reactivity in populations with limited mindfulness meditation experience (Baer et al., 2008). Furthermore, observing may not independently promote adaptive emotion regulation (Desrosiers et al., 2013). In addition, studies examining interactions among FFMQ facets suggest that the effects of observing depend on its combination with other acceptance components. For example, Eisenlohr-Moul et al. (2012) demonstrated that observing predicted lower substance use only when accompanied by high levels of non-reactivity. Taken together, these findings suggest that observing may not function effectively in isolation and may contribute to reduced Internet addiction only when combined with acceptance-oriented facets such as non-judging or acting with awareness.

### 4.1 Implications for mindfulness-based interventions

Our results show that Internet addiction is associated with the mindfulness facets of non-judging and acting with awareness. In particular, acting with awareness impacts Internet addiction even after controlling for other elements of mindfulness. These findings suggest the possibility of developing more effective interventions for Internet addiction by focusing training on non-judging and on acting with awareness, particularly the latter. Furthermore, standard mindfulness training takes a long time, lasting approximately 8 weeks. A meta-analysis of 98 randomized controlled trials reported an average attrition rate of approximately 29% in mindfulness interventions (Nam and Toneatto, 2016). Reducing emphasis on elements other than non-judging and acting with awareness may shorten training duration and improve participant retention. These modifications could reduce attrition rates and increase the number of participants who continue their training.

### 4.2 Limitations and future directions

There are several limitations to this study. First, the study used a cross-sectional design. As a result, its ability to elucidate causal relationships is limited. Therefore, future studies need to verify the effects of changes in acting with awareness and non-judging on Internet addiction through longitudinal studies and intervention studies. Second, the sample consisted exclusively of undergraduate and graduate students aged 18–25, limiting the generalizability of the findings to other populations, such as adolescents, older adults, and individuals with clinically significant Internet addiction. Moreover, the study did not focus on specific groups of participants by including an addicted group. As a result, future studies targeting broader age groups of participants and more serious addictions are needed to increase the generalizability of these results. Third, the regression model did not include psychological variables known to be associated with Internet addiction, such as depression (Alkan et al., 2025). Future research should control for these potential confounding variables. Fourth, the proportion of variance in Internet addiction explained by the model was modest, indicating that a substantial portion of the variance remains unaccounted for. Although acting with awareness emerged as an important predictor, other psychosocial factors, including anxiety, stress, loneliness, and impulsivity, have also been shown to be strongly associated with Internet addiction (Samanta et al., 2024). Future studies should therefore incorporate these variables to provide a more comprehensive understanding of Internet addiction.

### 4.3 Conclusion and recommendations

In conclusion, this cross-sectional study showed that the mindfulness facet of acting with awareness was uniquely and negatively associated with Internet addiction tendencies among university students. Given the cross-sectional design and the absence of some relevant covariates, the findings should be interpreted with caution. Nevertheless, they suggest that mindfulness-based interventions targeting acting with awareness may help mitigate problematic Internet use. Future longitudinal and clinical studies are needed to further examine and validate these findings.

## Data Availability

The raw data supporting the conclusions of this article will be made available by the authors, without undue reservation.

## Author’s note

This manuscript is an expanded and revised version of the first author’s master’s thesis completed during the 2025 academic year.
